# Melatonin alleviates chronic stress-induced hippocampal microglia pyroptosis and subsequent depression-like behaviors by inhibiting Cathepsin B/NLRP3 signaling pathway in rats

**DOI:** 10.1038/s41398-024-02887-y

**Published:** 2024-03-27

**Authors:** Zhicheng Gao, Kangxin Luo, Yulin Hu, Yunqian Niu, Xinchao Zhu, Shoujun Li, Haiyang Zhang

**Affiliations:** College of Veterinary Medicine, South China Agricultural University, Guangzhou, Guangdong Province 510642 PR China

**Keywords:** Molecular neuroscience, Pharmacodynamics

## Abstract

Melatonin improves chronic stress-induced hippocampal damage and depression-like behaviors, but the mechanism needs further study. This study was to explore the mechanism of melatonin inhibiting microglia pyroptosis. In virtro experiments, melatonin improved corticosterone-induced the ultrastructure and microstructure damage of HAPI cells by inhibiting pyroptosis, thereby increasing cell survival rate. Protein-protein interaction network and molecular autodocking predicted that Cathespin B might be the target of melatonin inhibition of NLRP3-mediated pyroptosis. Melatonin inhibited corticosterone-induced Cathespin B expression. Both Cathepsin B inhibitor CA-074Me and NLRP3 knockout inhibited the HAPI cells pyroptosis. Similarly, melatonin inhibited Cathepsin B agonist Pazopanib-induced activation of Cathepsin B/NLRP3 signaling pathway and HAPI cells pyroptosis. In vivo studies, melatonin inhibited chronic restraint stress (CRS)-induced activation of Cathepsin B/NLRP3 signaling pathway and alleviated hippocampal microglia pyroptosis in rats. Inhibition of microglia pyroptosis improved CRS-induced depression-like behaviors of rats. In addition, inhibition of Cathepsin B and NLRP3 alleviated hippocampal pyroptosis. Melatonin inhibited Pazopanib-induced activation of Cathepsin B/NLRP3 signaling pathway and hippocampal pyroptosis. These results demonstrated that melatonin could alleviate CRS-induced hippocampal microglia pyroptosis by inhibiting Cathepsin B/NLRP3 signaling pathway, thereby improving depression-like behaviors in rats. This study reveals the molecular mechanism of melatonin in the prevention and treatment of chronic stress-related encephalopathy.

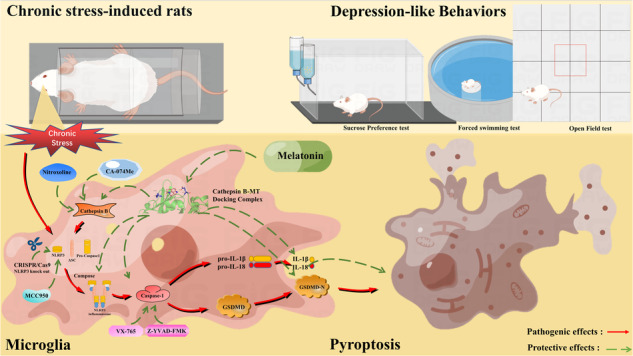

## Introduction

Depression is a mental disorder with significant global health implications and high rates of morbidity. The incidence of depression worldwide has increased from 172 million in 1990 to 258 million in 2017 [1, 2]. Chronic stress is considered a significant contributor to depression [3]. Studies indicate that chronic stress can result in excessive activation of the hypothalamic-pituitary-adrenal (HPA) axis [4]. The hippocampus plays a crucial role in regulating emotions and has a large number of glucocorticoid receptors. The hyperactivity of the HPA axis along with elevated levels of glucocorticoids can lead to hippocampal toxicity, resulting in depression [5]. Studies have shown that excessive glucocorticoids can induce microglia proliferation in the hippocampus, ultimately resulting in neuroinflammation [6]. However, the role of microglia in chronic stress-induced hippocampal damage remains to be further elucidated.

Microglia cells are the main innate immune cells in the brain [7]. Microglia become activated either by direct stimulation from an external pathogenic agent or in response to neuronal damage [8]. Under normal physiological conditions, microglia activation can effectively eliminate invading pathogens and secrete neurotrophic factors to regulate the stability of the microenvironment. While under pathological conditions, over-activated microglia secrete a large number of inflammatory cytokines, causing neuroinflammation and eventually leading to severe nerve damage [9]. Studies have also shown that overactivation of microglia is a major cause of depression [10]. Previous studies in our lab have shown that excessive activation of microglia caused by chronic stress is related to pyroptosis [11].

Pyroptosis is a lytic and inflammatory type of programmed cell death that is usually triggered by inflammasomes and ultimately executed by Gasdermin proteins [12]. During pyroptosis, the integrity of the cell membrane is destroyed and a large number of pro-inflammatory cytokines are released, leading to a strong inflammatory response that develops rapidly [13]. Classic Nod-like receptor protein 3 (NLRP3) inflammasome consists of NLRP3 protein, apoptosis-related spot-like protein containing CARD (ASC), and caspase-1. The NLRP3 inflammasome receives upstream signals to activate and produce Cleaved caspase-1, which transmits the signal to the subsequent executive protein Gasdermin D (GSDMD) [14]. GSDMD is cleaved to expose the N-terminal. GSDMD-N can specifically bind to and lysis the phospholipids of cell membranes to destroy the integrity of cell membranes [15]. At the same time, Cleaved caspase-1 causes inflammatory cytokines to mature and release in large quantities, eventually leading to a severe inflammatory response [16]. Studies have shown that the NLRP3 inflammasome plays an important role in the occurrence of depression [17]. Previous studies in our labs have shown that specific mechanism of chronic stress-induced pyroptosis of hippocampal microglia is closely related to NLRP3 inflammasome [11]. Therefore, inhibition of NLRP3 inflammasome-mediated microglia pyroptosis is like to be a treatment strategy for the chronic stress-induced hippocampal damage, but its therapeutic agents and protective mechanisms remain to be further studied.

Melatonin (MT) is an endogenous neurohormone that is widely used as a dietary supplement because of its healthcare function [18]. MT has various physiological functions such as anti-inflammatory, antioxidant, governing sleep, and relieving anxiety [19]. Because of its antioxidant capacity, MT has a protective effect on cardiovascular disease, diabetes, and other diseases [20]. Moreover, MT has significant effects on apoptosis, angiogenesis, tumor inhibition, and anti-proliferation of various tumor cells [21]. Studies have shown that MT can cross the blood-brain barrier and is involved in various neuroprotective actions on the brain [22]. MT also alleviates the overactivation of microglia, reduces nerve damage, and play a neuroprotective role [23]. Previous studies demonstrated that MT could alleviate lipopolysaccharides (LPS)-induced NLRP3 inflammasome formation and adipocyte pyroptosis, ultimately improving adipose tissue inflammation in mice [24]. Studies by our lab have shown that MT can improve chronic stress-induced neuroinflammation and apoptosis [25]. However, whether MT can inhibit microglia pyroptosis induced by chronic stress and its therapeutic targets and mechanisms remain unclear. In our previous studies, we confirmed that Cathepsin B as a potential regulator of NLRP3. Moreover, chronic stress can aggravate hippocampal microglia pyroptosis by activating the Cathepsin B/NLRP3 signaling pathway [11]. We, therefore, hypothesize that MT alleviates chronic stress-induced hippocampal microglia pyroptosis and subsequent depression-like behaviors by inhibiting the Cathepsin B/NLRP3 signaling pathway.

To verify this theoretical hypothesis, we first demonstrated that MT alleviates corticosterone (CORT)-induced rat microglia (HAPI cells) pyroptosis by inhibiting the Cathepsin B/NLRP3 signaling pathway using molecular docking technique, Cathepsin B agonist Pazopanib, Cathepsin B inhibitor CA-074Me, caspase-1 inhibitor Z-YVAD-FMK and CRISPR/Cas9 gene knockout technique in vitro. Subsequently, a chronic restraint stress (CRS) rat model and specific inhibitors and/or agonists were used. In animal studies, by using Cathepsin B agonist Pazopanib, Cathepsin B inhibitor Nitroxoline, NLRP3 inhibitor MCC950, and caspase-1 inhibitor VX-765, MT has been demonstrated to alleviate chronic stress-induced hippocampal microglia pyroptosis and subsequent depression-like behaviors by inhibiting Cathepsin B/NLRP3 signaling pathway. This study provides new therapeutic ideas and targets for chronic stress-related encephalopathy.

## Materials and methods

### Cell culture and drug treatments

The HAPI cells were obtained from the Henan Engineering Technology Research Center of Industrial Microbial Strains. The HAPI cells were authenticated and tested for mycoplasma contamination recently. HAPI cells were cultivated in DMEM medium (Gibco, Invitrogen, USA) supplemented with 10% FBS (BI) and 100 U/ml penicillin-streptomycin and were incubated at 37 °C in a humidified incubator (Thermo, USA) with 5% CO_2_. A concentration of 5 × 10^4^ cells/mL of HAPI cells was seeded in 6-well plates (*n* = 6) or 5 × 10^3^ cells/mL of HAPI cells was seeded 96-well plates (*n* = 6) before treatment for 24 h.

Cells were divided into nine groups based on different treatments randomly. In Control (CON) group, cells were incubated in complete medium without any treatment. In CORT group, cells were incubated in complete medium containing 40 μM CORT (≥98%, Yuanye Bio-Technology Co., Ltd, Shanghai, China) for 24 h. In MT + CORT group, cells were pretreated with complete medium containing 75 μM MT (Sigma-Aldrich, USA) for 30 min before being co-incubated with 40 μM CORT for 24 h. In MT group, cells were incubated with complete medium containing 75 μM MT for 24 h. In Z-YVAD-FMK + CORT group, cells were pretreated with 10 μM Z-YVAD-FMK (Selleck. cn, Shanghai, China) for 30 min before the addition of 40 μM CORT for 24 h. In CA-074Me + CORT group, cells were pretreated with 100 μM CA-074Me (Selleck. cn, Shanghai, China) for 2 h before the addition of 40 μM CORT for 24 h. In sgNLRP3 + CORT group, NLRP3 knockout cells were incubated in complete medium containing 40 μM CORT for 24 h. In Pazopanib group, cells were incubated in complete medium containing 20 μM Pazopanib (Selleck. cn, Shanghai, China) for 24 h. In Pazopanib + MT group, cells were pretreated for 30 min in complete medium containing 75 μM MT before being co-incubated with 20 μM Pazopanib for 24 h. In Z-YVAD-FMK + Pazopanib group, cells were pretreated with 10 μM Z-YVAD-FMK for 30 min before being co-incubated with 20 μM Pazopanib for 24 h. The dosage of Pazopanib [26], CA-074Me [26], and Z-YVAD-FMK [27] were based on previous studies. CORT and MT were dissolved in DMSO at a final concentration not exceeding 0.1%.

### CRISPR/Cas9 mediated genome editing

NLRP3 knockout in HAPI cells was established using CRISPR/Cas9 gene editing technology as our previously study [11]. In brief, the pX459-NLRP3-KO plasmid was constructed and NLRP3 had the following CRISPR target sites: ACGCTAATGATCGACTTCAA. In six-well plates, HAPI cells were seeded at a density of 1 × 10^5^ cells/well and transfected with 500 ng of pX459-NLRP3-KO plasmid using 1.5 μL of Lipofectamine^TM^ 2000 (Invitrogen, Garlsbad, CA, USA) in Opti-MEM (Thermo Fisher Scientific). Fresh complete serum medium was used to terminate the transfection after 6 h. The cells were screened with 1 mg/mL purinomycin and 10 μg doxycycline (LEAGENE, Beijing, China) on the fifth day after transfection to generate stable monoclonal cell lines. Western blot was used to confirm NLRP3 expression.

### Cells viability assay

HAPI cells were seeded at a density of 5 × 10^3^ cells/well in 96-well plates and treated during 24 h to different concentrations of CORT (0 nM, 100 nM, 1 μM, 10 μM, 50 μM, 100 μM) and MT (0 μM, 25 μM, 50 μM, 75 μM, 100 μM, 125 μM, 150 μM, 175 μM, 200 μM) and for 24 h. Cells viability was determined using the cell counting kit-8 assay (Beyotime, Shanghai, China) according to the manufacturer’s instructions.

### Inflammatory cytokines and lactate dehydrogenase (LDH) release assay

Interleukin-1β (IL-1β) and interleukin-18 (IL-18) levels in HAPI cell culture supernatants were determined using corresponding ELISA assay kits (Boster Biological Technology Co., Ltd., Wuhan, China) according to the manufacturer’s instructions, respectively. LDH was measured in the culture supernatants of HAPI cells using an LDH assay kit (Beyotime, Jiangsu, China) as directed by the manufacturer.

### Flow cytometry analysis

The cellular death of HAPI cells were measured using Annexin V-FITC/PI analysis kit (Bioss, Beijing, China) according to the manufacturer’s instructions. In brief, the cells were stained with 10 μL Annexin V-FITC and 10 μL PI for 15 min at room temperature under dark conditions. Annexin V-FITC and PI fluorescence were detected with a flow cytometry (BD FACSVerse™ Flow Cytometer, BD Biosciences, USA) and data were analyzed using FlowJo software (Version 10.0; Three Star).

### Protein-protein interaction (PPI) network analysis

The PPI network analysis process was performed in detail as we previously reported [11]. To put it simply, the STRING database (https://string-db.org/) was used to look for putative NLRP3 regulating proteins. The Kyoto Encyclopedia of Genes and Genomes (KEGG) pathway analysis was performed on all proteins in the PPI network (https://www.kegg.jp/) (Fig. A.1A).

### Molecular docking simulation

The computational docking technique was used to estimate the interaction between MT with Cathepsin B. AutoDock (https://autodock.scripps.edu/) was used to design the docking programs. The crystal structures of Cathepsin B were available from the Protein Data Bank (https://www.rcsb.org/) under accession numbers P00787. The ZINC database (https://zinc.docking.org/) was used to get the chemical structures of MT (ZINC57060). The best-fit pose of docked molecules, binding energy values, possible conformations, bond distances, and types of interactions were all predicted based on docking data.

### Animals and experimental groups

One hundred and twenty Wistar rats, half male and half female (six weeks of age, weight 200 ± 20 g) were obtained from the Center for Experimental Animals of Southern Medical University (approval number: SCXK 2021-0041). All of the animals were kept in the Animal Experiment Center of South China Agricultural University (approval number: SYXK 2022-0136) and allowed to acclimatize to a standard laboratory condition (temperature 22 ± 2 °C, humidity 50 ± 5%, 12 h light/dark cycle) for 7 days. Water and food were available ad libitum.

In order to determine the protective effect and mechanism of MT on CRS-induced hippocampal microglia pyroptosis in rats, 48 rats were randomly allocated into four groups (*n* = 12, half male and half female, 3 rats per cage). CON group: rats were not disturbed in their cages. CRS group: rats were held in special rat fixators for 21 days to experience restraint stress, from 9:00 AM to 3:00 PM each day. CRS + MT group: rats were given an intraperitoneally injection of MT (10 mg/kg dissolved in 0.2% alcohol-saline solution, Sigma Aldrich, USA) 30 min before the restraint stress daily for 21 days. ALC + CRS group: rats were given intraperitoneally injected 0.2% alcohol-saline solution intraperitoneally 30 min before being restrained daily for 21 days.

To determine whether hippocampal microglia pyroptosis is involved in chronic stress-induced depression-like behaviors in rats, 24 rats were randomly divided into two groups (*n* = 12, half male and half female, 3 rats per cage). VX-765 group: rats were administered VX-765 (50 mg/kg dissolved in 10% DMSO and 90% corn oil, Medchemexpress, USA) by intragastric gavage 30 min before restraint stress daily for 21 days. V + CRS group: rats were given the same volume solution of 10% DMSO and 90% corn oil by intragastric gavage 30 min before restraint stress daily for 21 days.

To confirm the molecular mechanisms by which MT alleviates chronic stress-induced microglia pyroptosis, 48 rats were randomly divided into 8 groups (*n* = 6, 3 rats per cage). Nitroxoline + CRS group: rats were injected intraperitoneally Nitroxoline (40 mg/kg dissolved in 5% DMSO in peanut oil, Selleck. cn, Shanghai, China) 30 min before restraint stress daily for 21 days. N + CRS group: rats were injected intraperitoneally the same volume of 5% DMSO in peanut oil 30 min before being restrained daily for 21 days. MCC950 + CRS group: rats were injected intraperitoneally MCC950 (10 mg/kg dissolved in normal saline, Selleck. cn, Shanghai, China) 30 min before restraint stress daily for 21 days. M + CRS group: rats were injected intraperitoneally the same volume of normal saline 30 min before being restrained daily for 21 days. Pazopanib group: rats were intraperitoneally injected Pazopanib (30 mg/kg dissolved in 2% Tween, 5% propylene glycol in 0.9% saline solution, Selleck. cn, Shanghai, China) daily for 21 days. P + CON group: rats were administered intraperitoneally the same volume of 2% Tween, 5% propylene glycol in 0.9% saline solution daily for 21 days. MT + Pazopanib group: rats were intraperitoneally injected 30 mg/kg Pazopanib and 10 mg/kg MT daily for 21 days. ALC + Pazopanib group: rats were intraperitoneally injected 30 mg/kg Pazopanib and the same volume of 0.2% alcohol-saline solution daily for 21 days.

During the restraint stress period, none of the rats was given food or water. The CRS procedure in detail was executed as our previously reported [28, 29]. The dosage and use of MT [30], VX-765 [31], Pazopanib [31], Nitroxoline [32], and MCC950 [33] were determined based on previous studies. The investigators were blinded to the group allocation during the experiment. In all cases of animal experimentation, the Animal Ethics Committee of South China Agricultural University, Guangzhou, China, approved the procedure (No. 2023f185).

### Behavioral tests

Depression-like behaviors such as autonomic activity, spatial exploration, anxiety, anhedonia, and despair behaviors of rats were fully evaluated by open field test, sucrose preference test, and forced swimming test. Detailed procedures of these behavioral tests have been reported in our studies [28, 29]. The investigators were blinded to the group allocation during the experiment.

### Histological and ultrastructural observations

The brains of the rats were rapidly removed and fixed in 10% formalin for 48 h after anesthesia and euthanasia. After that, the brain was then embedded in paraffin and chopped into 5 μm-thick slices. The slices were dewaxed and hydrated with an anhydrous ethanol concentration gradient. The slices were stained with hematoxylin and eosin staining according to the manufacturer’s instructions (Beyotime, Jiangsu, China). Finally, the morphological structure of the hippocampus in the slices was observed using pathological optical microscopy (Nikon 80i, Nikon, Japan). ImageJ software was utilized for quantitative analysis at 400× magnification.

The rat hippocampus was carefully peeled out and cut into 1 mm^3^ cubes, then fixed for 48 h at 4 °C in 2.5% glutaraldehyde. The hippocampus cubes were properly washed in PBS before being fixed for 1 h in 1% osmium tetroxide. The hippocampal cubes were then nested in resin after being dehydrated with ethanol at varied concentration gradients. The hippocampus-containing resin blocks were then cut into 70 nm thick slices and stained for 5 min with lead citrate. A transmission electron microscope (HT-7650, Hitachi Company, Japan) was used to examine the ultrastructure of the rat hippocampus.

### Triple-labeled immunofluorescent staining

Brain slices were blocked with 10% goat serum for 30 min before being incubated overnight at 4 °C with the first primary antibody anti-Iba-1 (Servicebio, Wuhan, China), followed by the first matching secondary antibody. the slices were then incubated with the second primary antibody anti-NLRP3 (Servicebio, Wuhan, China) and the second secondary antibody, followed by the third primary antibody anti-Cleaved caspase-1 (Servicebio, Wuhan, China) and the third secondary antibody. DAPI was used to counter-stain HAPI cells and brain slides. A fluorescent microscope was used to obtain the pictures at 400× magnification. Detailed procedure of triple-labeled immunofluorescent staining has been reported in our study [29].

### Western blot analysis

Total proteins were extracted from HAPI cells and the hippocampus using Western and IP cell lysis buffer or RIPA buffer (Beyotime, Shanghai, China) and detected as previously described [28, 29], respectively. In brief, the protein sample (20–30 μg per lane) was separated by SDS-PAGE. After then, the target protein was transferred to PVDF membranes. The membranes were incubated overnight at 4 °C with primary antibodies of Cathepsin B (Abcam, ab214428), NLRP3 (Abcam, ab263899), ASC (Abcam, ab180799), Cleaved caspase-1 (Abcam, ab207802), GSDMD-N (Abcam, ab219800), IL-1β (Bioss, bs-25615R), IL-18 (Bioss, bs-4986R) and GAPDH (ZSGB-BIO, TA-08), respectively. The membranes were incubated for 2 h at room temperature with the matching secondary antibodies. ECL western blot detection system (Tanon Science & Technology Co. Ltd., Shanghai, China) was used to visualize the target protein bands, which were quantified using ImageJ software.

### Quantitative real-time PCR analysis

Total RNA was extracted from HAPI cells using HiPure Universal RNA Mini Kit (Magenbio, Guangzhou, China) and reverse transcribed using GoScript^TM^ Reverse Transcription Mix, Oligo (dT) (Promega, Shanghai, China) following the manufacturer’s instructions. Roche 480 Real-Time PCR System (Roche, CH) was used to perform quantitative real‐time PCR using the IQ SYBR Green Supermix reagent (Bio‐Rad, San Diego, CA). As the internal control, *GAPDH* was utilized. Relative mRNA expression of the target genes was calculated using the 2^−ΔΔCt^ method [34]. The primer sequences are shown in Table 1.

**Table 1:** Primer sequence and amplification length of destination fragment.

Gene	Number	Upstream and downstream primer sequence	Product length (bp)
*Panx1*	NM_001270548.1	F: 5′-GCTGCACAAGGTAATAATGAGTCT-3′ R: 5′-AGGGCGTACACTAGGAGGTT-3′	333
*P2rx7*	NM_019256.2	F: 5′-GCTGAGAATCGGTGTGCTTTC-3′ R: 5′-CTGCAACGCCTTTGACCTTG-3′	316
*Mavs*	NM_001005556.1	F: 5′-CTTTAGCAAGCAGTCCATCTCT-3′ R: 5′-TGCTTGTAGGAAGCCCGTAA-3′	218
*Txnip*	NM_001008767.2	F: 5′-TAGTGATTGGCAGCAGGTCG-3′ R: 5′-CTCAGTGTAAGTGGGCGGAG-3′	260
*Cathepsin B*	NM_022597.2	F: 5′-CAGGCTGGACGCAACTTCTA-3′ R: 5′-CAGGTAAGCAGGTCCTCAGC-3′	299
*Nek7*	NM_006249926.3	F: 5′-ATGGATGAACAACCACAAGGAAT-3′ R: 5′-TGGAACTCCATCCAGGAGACA-3′	177
*GAPDH*	NM_017008.4	F: 5′-AGTGCCAGCCTCGTCTCATA-3′ R: 5′-GATGGTGATGGGTTTCCCGT-3′	248

### Statistical analysis

All data was analyzed using SPSS 22.0 software (SPSS, IL, USA). Multiple groups of data were analyzed and compared using one-way analysis of variance and the Tukey post hoc test. The data were estimated for each group. The data conformed to the normal distribution the tests. The variance/standard deviation is similar between the groups that are being statistically compared. The mean ± standard deviation (SD) was used to express the data. When *P* < 0.05, statistical differences were judged significant. When *P* < 0.01, statistical differences were judged extremely significant.

## Results

### MT attenuates CORT-induced HAPI cells damage

**Fig. 1:**
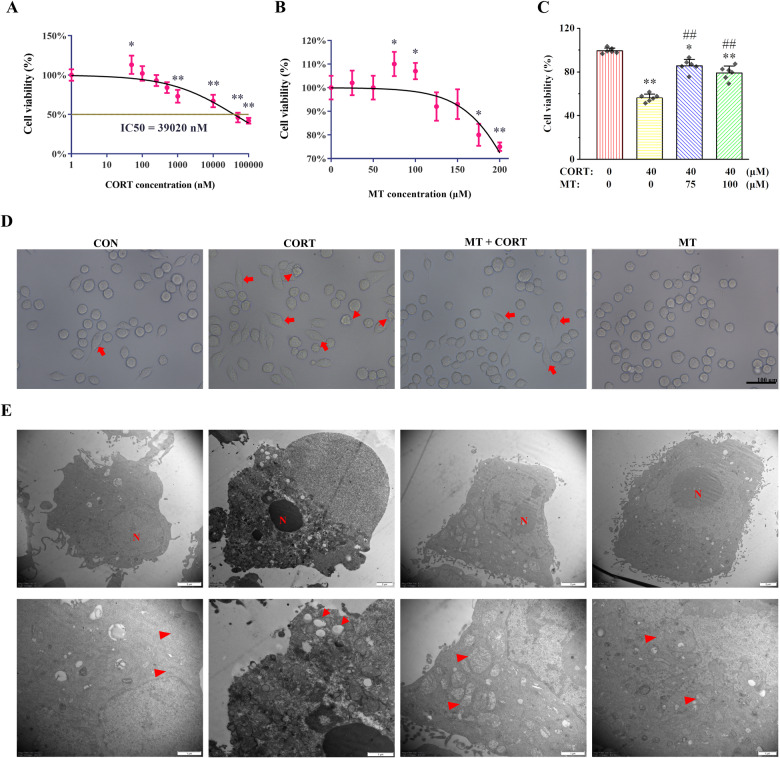
Effects of MT on cell viability, cell morphology, and ultrastructure in HAPI cells treated by CORT. **A** HAPI cells viability after treatment with different concentrations of CORT. **B** HAPI cell viability after treatment with different concentrations of MT. **C** The effect of 75 and 100 μM MT on the cell viability of 40 μM CORT-treated HAPI cells. **D** The representative morphology images of HAPI cells; 200× magnification; scale bars = 100 µm. The red thick-tailed arrows indicate that some cells have been deformed and are fusiform. The red thin-tailed arrows indicate that some cell membranes have broken. **E** The representative ultrastructural images of HAPI cells. See the lower left corner of each picture for specific magnification; Scale = 2 μm or 1 μm. The red “N” indicates nucleus. The red triangle represents normal organelles (mitochondria, Golgi bodies, etc.) in the cytoplasm. The red thin-tailed arrow indicates mitochondrial cavitation. All data are presented as the mean ± SD (*n* = 6). ^*^P < 0.05 and ^**^P < 0.01 versus 0 μM CORT and 0 μM MT group. ^##^*P* < 0.01 versus 40 μM CORT and 0 μM MT group.

The cytotoxicity of different concentrations of CORT to HAPI cells is shown in Fig. 1A. After analysis and calculation, the IC50 of HAPI cells for CORT was 39,020 nM. Therefore, 40 μM (approximately equal to IC50) CORT was selected for subsequent cell experiments. At this concentration, the survival rate of HAPI cells decreased significantly (*P* < 0.01). The effect of MT on HAPI cells viability is shown in Fig. 1B. For MT concentrations, the survival rate of HAPI cells was significantly increased at 75 and 100 μM, especially at 75 μM. The effect of MT on the viability of CORT-treated HAPI cells was shown in Fig. 1C. Both 75 and 100 μM MT significantly increased the survival rate of HAPI cells treated with 40 μM CORT (Fig. 1C, *P* < 0.01). These results suggest that CORT increases cytotoxicity, while MT improves cells viability. Based on these results, the concentration selection of CORT and MT in this experiment was 40 and 75 μM, respectively, for subsequent experiments.

The morphology of HAPI cells is shown in Fig. 1D. The HAPI cells in CON group and MT group were round or oval, with good refraction and good growth state. In CORT group, some cells were spindle-shaped or irregular (The red thick-tailed arrows), poor refraction, poor growth, and even rupture (The red thin-tailed arrows). However, after MT treatment, the above situation was significantly improved. Transmission electron microscopy showed that cells were round with complete cell structure, uniform distribution of nuclear chromatin, and clear mitochondria (The red triangle) in CON and MT groups. In CORT group, cells were deformed, cell membrane ruptured, the nuclear chromatin concentrated, and the mitochondria vacuolated (The red thin-tail arrows). In MT + CORT group, the ultrastructural damage was reversed obviously (Fig. 1E). These results suggest that MT ameliorates the pathological structural damage of HAPI cells induced by CORT.

### MT attenuates CORT-induced HAPI cells pyroptosis by inhibition NLRP3 inflammasome activation

**Fig. 2:**
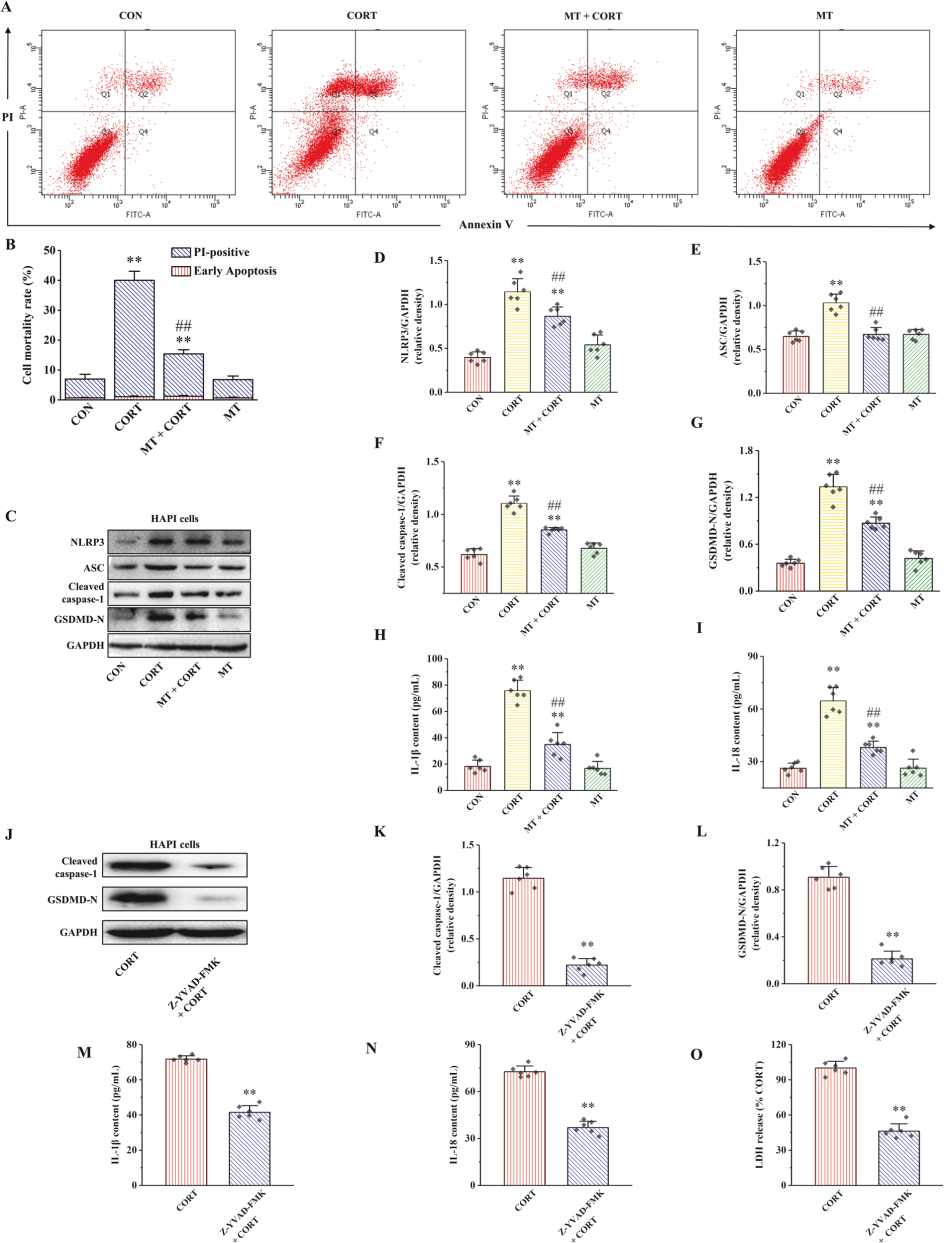
Effects of MT or Z-YVAD-FMK on NLRP3 inflammasome activation and pyroptosis in HAPI cells treated by CORT. **A** The death of HAPI cells was detected by flow cytometry. **B** The PI-positive HAPI cells rate. **C** The relative protein expression of NLRP3, ASC, Cleaved caspase-1, and GSDMD-N. **D**–**G** Proteins quantitative analysis of NLRP3, ASC, Cleaved caspase-1, and GSDMD-N. **H**, **I** The content of IL-1β, and IL-18. **J** Relative protein expression of Cleaved caspase-1 and GSDMD-N. **K**, **L** Proteins quantitative analysis of Cleaved caspase-1 and GSDMD-N. **M**–**O** The contents of IL-1β, IL-18, and LDH. All data are presented as the mean ± SD (*n* = 6). ^*^P < 0.05 and ^**^P < 0.01 versus CON group. ^##^*P* < 0.01 versus CORT group.

The death rate of HAPI cells in different groups was detected by flow cytometry, as shown in Fig. 2A, B. Compared with CON group, the rate of PI-positive cells in CORT group was significantly increased (*P* < 0.01). Compared with CORT group, the positive rate of PI cells in MT + CORT group was significantly decreased (*P* < 0.01). The pyroptosis related protein expression levels of NLRP3, ASC, Cleaved caspase-1, and GSDMD-N were greatly increased in CORT group compared with CON group (*P* < 0.01), while were significantly declined in MT + CORT group compared with CORT group (Fig. 2C–G, *P* < 0.01). Compared with CON groups, the content of IL-1β and IL-18 in the supernatants of HAPI cells were significantly increased in CORT group (*P* < 0.01), while was significantly lower in MT + CORT group than in CORT group (Fig. 2H, I, *P* < 0.01).

In addition, the effect of pyroptosis inhibitor Z-YVAD-FMK on CORT-induced HAPI cells pyroptosis is shown in Fig. 2J–O. Compared with CORT group, the protein expressions of Cleaved caspase-1 and GSDMD-N were significantly decreased in Z-YVAD-FMK + CORT group (Fig. 2J–L, *P* < 0.01). Similarly, the contents of IL-1β, IL-18, and LDH in the supernatants of HAPI cells were reduced in Z-YVAD-FMK + CORT group compared with CORT group (Fig. 2M–O, *P* < 0.01).

Taken together, these results suggest that CORT induces HAPI cells pyroptosis, while MT can improve CORT-induced HAPI cells pyroptosis by inhibiting NLRP3 inflammasome activation.

### Cathepsin B is a potential target of MT regulation of NLRP3

**Fig. 3:**
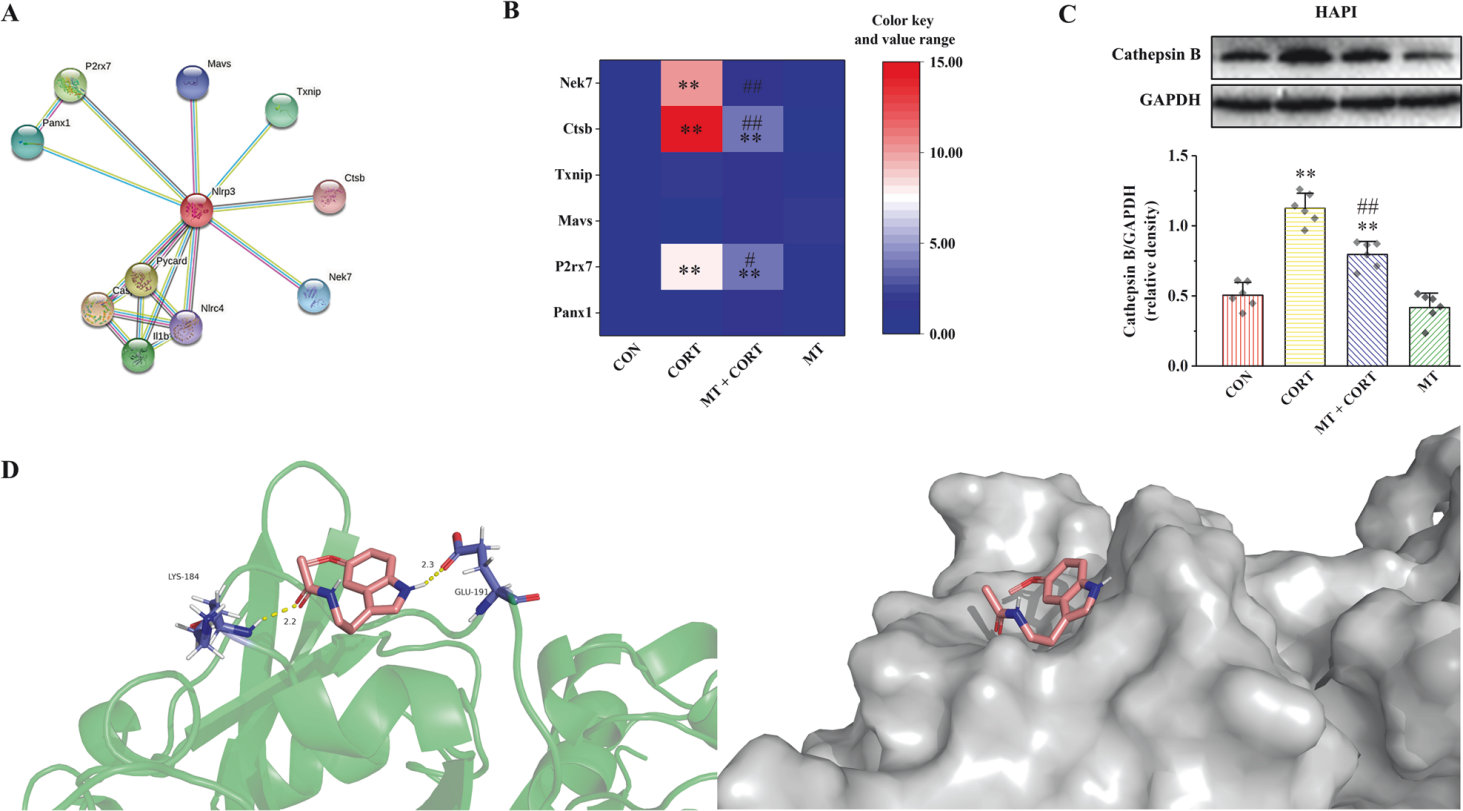
Cathepsin B is a potential target of MT-regulated NLRP3 inflammasome activation and pyroptosis in HAPI cells. **A** Potential regulatory proteins of NLRP3 were predicted by the PPI network. **B** The relative mRNA expression of NLRP3 potential regulatory proteins. **C** Expression of Cathepsin B protein in HAPI cells. **D** Simulation model of molecular docking between MT and Cathepsin B. Ctsb Cathepsin B. All data are presented as the mean ± SD (*n* = 6). ^**^P < 0.01 versus CON group. ^#^*P* < 0.05 and ^##^*P* < 0.01 versus CORT group. ^&&^*P* < 0.01 versus MT group.

PPI network analysis results suggest that six proteins include Panx1, P2rx7, MAVS, TXNIP, Cathepsin B, and Nek7 are the potential regulators of NLRP3 (Fig. 3A). KEGG pathway enrichment analysis showed that these genes were enriched in nod like receptor signaling pathway, NLRP3 inflammasome and mechanical stimulation response pathway (Fig. A.1B). Moreover, the results of the mRNA expression of these potential NLRP3 regulators are shown in Fig. 3B. Among these six proteins, the mRNA expression of *Cathepsin B*, *Nek7* and *P2rx7* were significantly increased in CORT group compared with CON group, while were obviously decreased in MT + CORT group compared with CORT group (*P* < 0.05 and *P* < 0.01). Notably, *Cathepsin B* showed the most significant change. Furthermore, compared with CON group, the protein expression of Cathepsin B was significantly increased in CORT group, while MT + CORT group was significantly lower than CORT group (Fig. 3C, *P* < 0.01).

In order to analyze any possible interaction and binding mechanism of MT with Cathepsin B, AutoDock tools were used to successfully construct simulation models of MT-Cathepsin B complexes by protein macromolecular and ligand docking (Fig. 3D). Guided by the docking simulation of the binding conformation, two hydrogen bonds are formed between MT and Cathepsin B. MT was able to bind with Cathepsin B at residues LYC-184 and GLU-191 with a binding-energy value of −5.68 kcal/mol. Taken together, these results suggest that Cathepsin B is a potential target of MT in the regulation of NLRP3.

### Both CA-074Me and sgNLRP3 alleviate CORT-induced HAPI cells pyroptosis by inhibiting Cathepsin B/NLRP3 signaling pathway

**Fig. 4:**
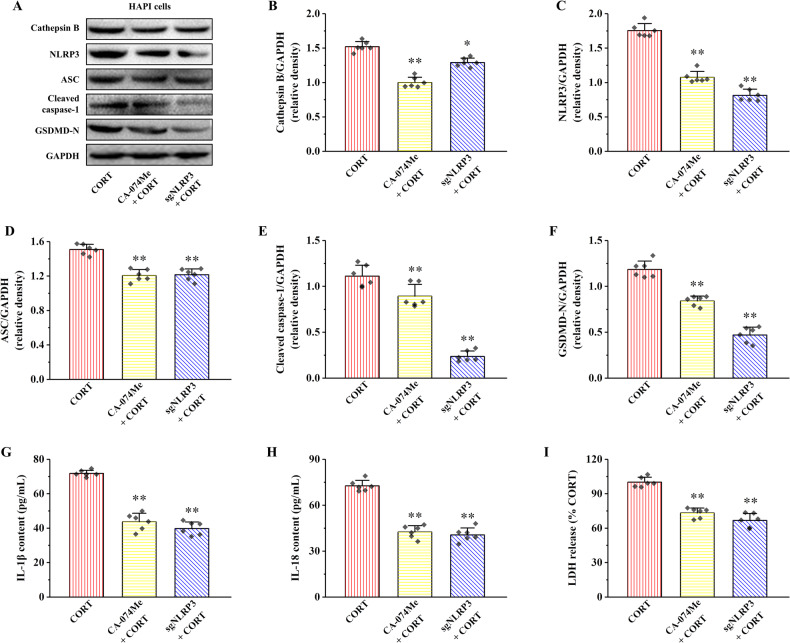
Effects of CA-074Me and sgNLRP3 on Cathepsin B expression, NLRP3 inflammasome activation and pyroptosis in HAPI cells treated by CORT. **A** The relative protein expression of Cathepsin B, NLRP3, ASC, Cleaved caspase-1, and GSDMD-N. **B**–**F** Proteins quantitative analysis of Cathepsin B, NLRP3, ASC, Cleaved caspase-1, and GSDMD-N. **G**–**I** The contents of IL-1β, IL-18, and LDH. All data are presented as the mean ± SD (*n* = 6). ^**^P < 0.01 versus CORT group.

Compared with CORT group, the protein expressions of Cathepsin B, NLRP3, ASC, Cleaved caspase-1, and GSDMD-N were significantly decreased in CA-074Me and sgNLRP3 groups (Fig. 4A–F, *P* < 0.05 and *P* < 0.01). The contents of IL-1β, IL-18, and LDH in CA-074Me and sgNLRP3 groups were also significantly reduced compared with CORT group (Fig. 4G–I, *P* < 0.01). These results suggest that both CA-074Me and sgNLRP3 alleviate CORT-induced HAPI cells’ pyroptosis by inhibiting Cathepsin B/NLRP3 signaling pathway.

### MT relieves Pazopanib-induced HAPI cells pyroptosis by Cathepsin B/NLRP3 signaling pathway

**Fig. 5:**
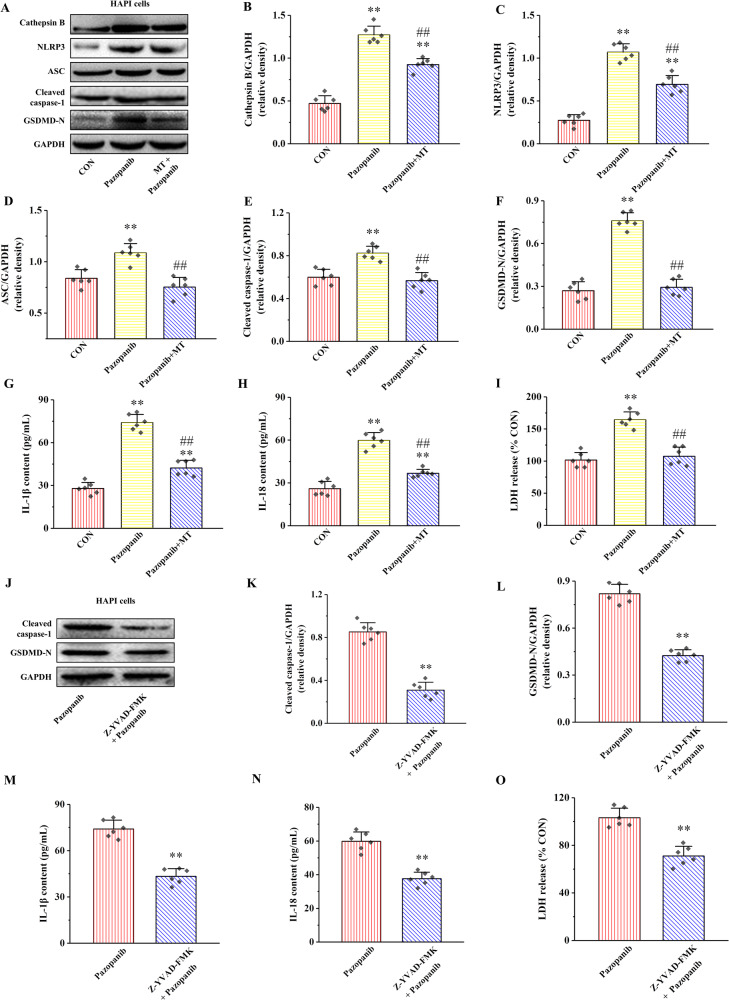
Effects of MT or Z-YVAD-FMK on Cathepsin B expression, NLRP3 inflammasome activation and pyroptosis in HAPI cells treated by Pazopanib. **A** The relative protein expression of Cathepsin B, NLRP3, ASC, Cleaved caspase-1, and GSDMD-N. **B**–**F** Proteins quantitative analysis of Cathepsin B, NLRP3, ASC, Cleaved caspase-1, and GSDMD-N. **G**–**I** The contents of IL-1β, IL-18, and LDH. All data are presented as the mean ± SD (*n* = 6). ^**^P < 0.01 versus CON group. ^##^*P* < 0.01 versus Pazopanib group. **J** The relative protein expression of Cleaved caspase-1 and GSDMD-N. **K**, **L** Proteins quantitative analysis of Cleaved caspase-1 and GSDMD-N. **M**–**O** The contents of IL-1β, IL-18, and LDH. All data are presented as the mean ± SD (*n* = 6). ^**^P < 0.01 versus Pazopanib group.

The proteins expression of Cathepsin B, NLRP3, ASC, Cleaved caspase-1, and GSDMD-N in Pazopanib groups were significantly increased relative to those in CON group, whereas these protein levels were clearly declined in Pazopanib + MT compared with Pazopanib groups(Fig. 5A–F, *P* < 0.01). Likewise, the content of IL-1β, IL-18, and LDH were significantly increased in Pazopanib group compared with CON group, while the contents of IL-1β, IL-18, and LDH were obviously decreased in Pazopanib + MT compared with Pazopanib group (Fig. 5G–I, *P* < 0.01).

Moreover, the effect of Z-YVAD-FMK on Pazopanib-induced HAPI cells pyroptosis is shown in the Fig. 5J–O. Compared with Pazopanib group, the protein expressions of Cleaved caspase-1 and GSDMD-N were significantly decreased in Z-YVAD-FMK + Pazopanib group (Fig. 5J–L, *P* < 0.01). Similarly, the contents of IL-1β, IL-18, and LDH in the supernatants were reduced in Z-YVAD-FMK + Pazopanib group compared with Pazopanib group (Fig. 5M–O, *P* < 0.01).

Taken together, these results suggest MT alleviates Pazopanib-induced pyroptosis by inhibiting Cathepsin B/NLRP3 signaling pathway in HAPI cells.

### MT alleviates CRS-induced hippocampal microglia pyroptosis by inhibiting Cathepsin B/NLRP3 signaling pathway

**Fig. 6:**
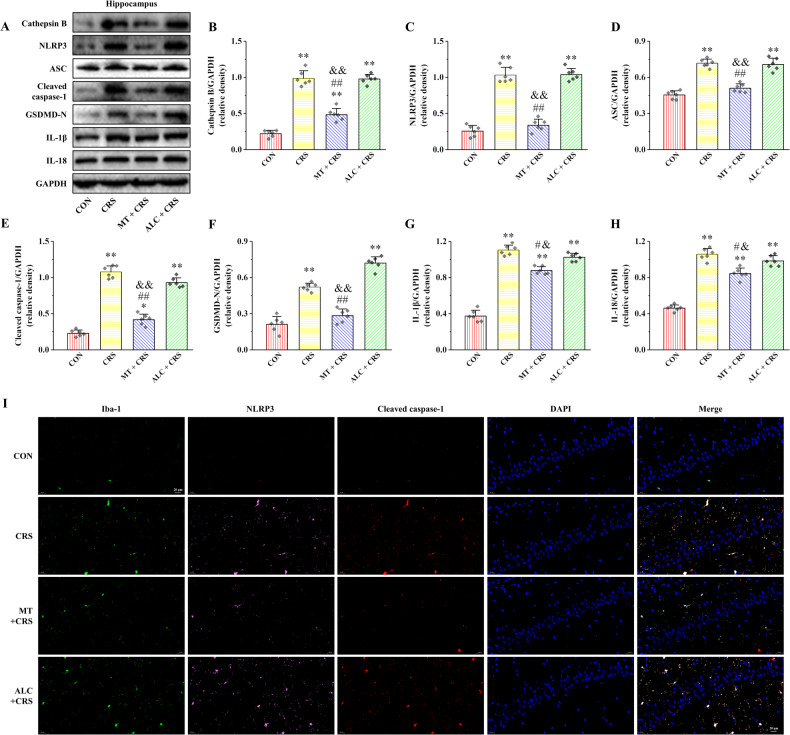
Effects of MT on hippocampal Cathepsin B expression, NLRP3 inflammasome activation and pyroptosis in CRS rats. **A** The relative protein expression of Cathepsin B, NLRP3, ASC, Cleaved caspase-1, GSDMD-N, IL-1β, and IL-18. **B**–**H** Proteins quantitative analysis of Cathepsin B, NLRP3, ASC, Cleaved caspase-1, GSDMD-N, IL-1β, and IL-18. **I** Representative Iba-1, NLRP3, and Cleaved caspase-1 triple-labeled immunofluorescent images in the hippocampus, 400× magnification; scale bars = 20 µm. All data are presented as the mean ± SD (*n* = 6). ^*^P < 0.05 and ^**^P < 0.01 versus CON group. ^#^*P* < 0.05 and ^##^*P* < 0.01 versus CRS group. ^&^*P* < 0.05 and ^&&^*P* < 0.01 versus ALC + CRS group.

The proteins expression of Cathepsin B, NLRP3, ASC, Cleaved caspase-1, GSDMD-N, IL-1β, and IL-18 were greatly increased in CRS and ALC + CRS groups compared with CON group (*P* < 0.01), while were significantly decreased in CRS + MT group compared with CRS and ALC + CRS groups (Fig. 6A–H, *P* < 0.05 and *P* < 0.01). Furthermore, the results of three-labeled immunofluorescence staining of Iba-1, NLRP3, and Cleaved caspase-1 in the hippocampus are shown in Fig. 6I. Compared with CON group, Iba-1, NLRP3, and Cleaved caspase-1 positive cells were obviously increased in the hippocampus of CRS and ALC + CRS groups, and co-localized expression of the three cells was observed. On the contrary, the Iba-1, NLRP3, and Cleaved caspase-1 positive cells in MT + CRS group was clearly reduced compared with CRS and ALC + CRS groups. These results suggest that MT can partially relieve hippocampal microglia pyroptosis by inhibiting Cathepsin B/NLRP3 signaling pathway of CRS rats.

### VX-765 improves hippocampal microglia pyroptosis and depression-like behaviors in CRS rats

**Fig. 7:**
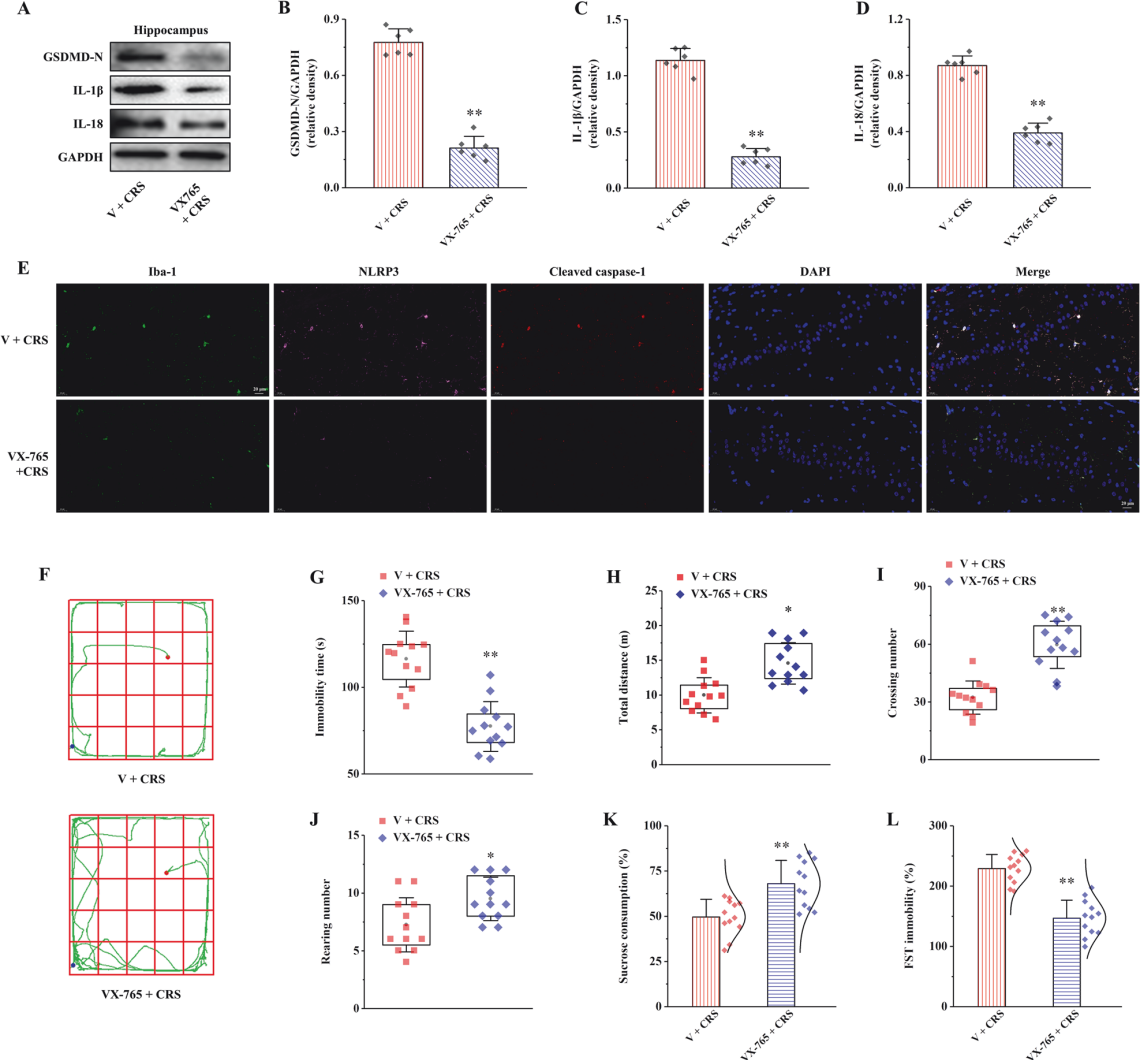
Effects of VX-765 on hippocampal NLRP3 inflammasome activation, pyroptosis and depression-like behaviors in CRS rats. **A** The relative protein expression of GSDMD-N, IL-1β, and IL-18. **B**–**D** Proteins quantitative analysis of GSDMD-N, IL-1β, and IL-18 (*n* = 6). **E** Representative Iba-1, NLRP3, and Cleaved caspase-1 triple-labeled immunofluorescent images in the hippocampus, 400× magnification scale bars = 20 µm (*n* = 6). **F** The movement trajectories of rats. The red dot represents the starting point of the rat’s movement, the green line represents the specific movement track of the rat, and the blue dot represents the ending point of the rat’s movement. **G** The immobility time of the rats in open field test. **H** The total distance of the rats traveled. **I** The number of times the rats crossed the grid lines. **J** The number of times the rats stood. **K** The sucrose consumption of the rats in a sucrose preference test. **L** The immobility time of the rats in forced swimming test (*n* = 12). All data are presented as the mean ± SD. ^##^*P* < 0.01 versus V + CRS group.

The proteins expression of GSDMD-N, IL-1β, and IL-18 in VX-765 + CRS group was significantly decreased compared with V + CRS group (Fig. 7A–D, *P* < 0.01). Moreover, compared with the V + CRS group, the co-localized expression of Iba-1, NLRP3, and caspase-1 in the hippocampus of rats in the VX-765 + CRS group was observed, but the number of positive cells was reduced (Fig. 7E).

The results of the open-field test of rats are shown in Fig. 7F–J. The movement locus of V + CRS group was mainly concentrated in the peripheral region. However, the motor trajectory of rats in VX-765 + CRS group tended to the middle region slightly than that in V + CRS group. Moreover, the immobility time was significantly reduced in VX-765 + CRS group compared with V + CRS group (Fig. 7G, *P* < 0.01). On the contrary, the total moving distance, times of crossing the grid and times of standing observation of rats in VX-765 + CRS group were significantly increased compared with V + CRS group (Fig. 7H–J, *P* < 0.05 and *P* < 0.01). In the sucrose preference test, the consumption of sucrose in VX-765 + CRS group was significantly higher than V + CRS group (Fig. 7K, *P* < 0.01). In the forced swimming test, the immobility time of rats in VX-765 + CRS group was significantly shorter than that in V + CRS group (Fig. 7L, *P* < 0.01). These results suggest that VX-765 can ameliorate hippocampal microglia pyroptosis and depression-like behaviors in CRS rats, such as disturbance of spontaneous motor activity, anhedonia, and increased desperate behaviors.

### Inhibition of Cathepsin B/NLRP3 signaling pathway alleviates CRS-induced hippocampal pyroptosis

**Fig. 8:**
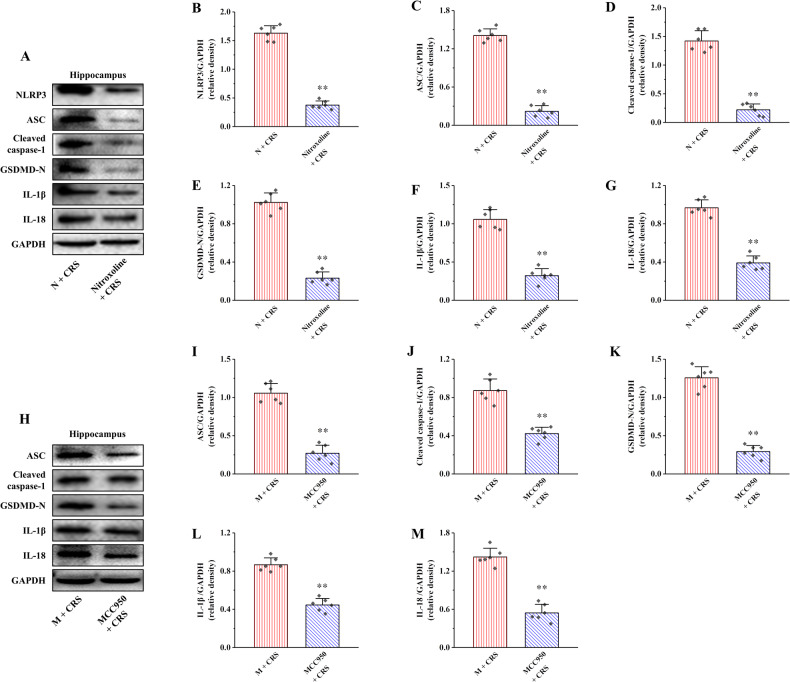
Effects of nitroxoline and MCC950 on hippocampal NLRP3 inflammasome activation and pyroptosis in CRS rats. **A**, **H** The relative protein expression of NLRP3, ASC, Cleaved caspase-1, GSDMD-N, IL-1β, and IL-18. **B** Proteins quantitative analysis of NLRP3. **C**, **I** Proteins quantitative analysis of ASC. **D**, **J** Proteins quantitative analysis of Cleaved caspase-1. **E**, **K** Proteins quantitative analysis of GSDMD-N. **F**, **L** Proteins quantitative analysis of IL-1β. **G**, **M** Proteins quantitative analysis of IL-18. All data are presented as the mean ± SD (*n* = 6). ^**^P < 0.01 versus N + CRS or M + CRS group.

To verify whether Cathepsin B/NLRP3 signaling pathways are involved in CRS-induced hippocampal microglia pyroptosis, Nitroxoline, and MCC950 were used to inhibit Cathepsin B and NLRP3, respectively. The proteins expression of NLRP3, ASC, Cleaved caspase-1, GSDMD-N, IL-1β and IL-18 in Nitroxoline + CRS group were significantly decreased relative to those in N + CRS group (Fig. 8A–G, *P* < 0.01). Similarly, the proteins expression of ASC, Cleaved caspase-1, GSDMD-N, IL-1β, and IL-18 were also clearly declined in MCC950 + CRS group compared with M + CRS group (Fig. 8H–M, *P* < 0.01). These results suggest that inhibition of Cathepsin B/NLRP3 signaling pathway can alleviate hippocampal pyroptosis in CRS rats.

### MT attenuates Pazopanib-induced hippocampal pyroptosis by inhibiting Cathepsin B/NLRP3 signaling pathway

**Fig. 9:**
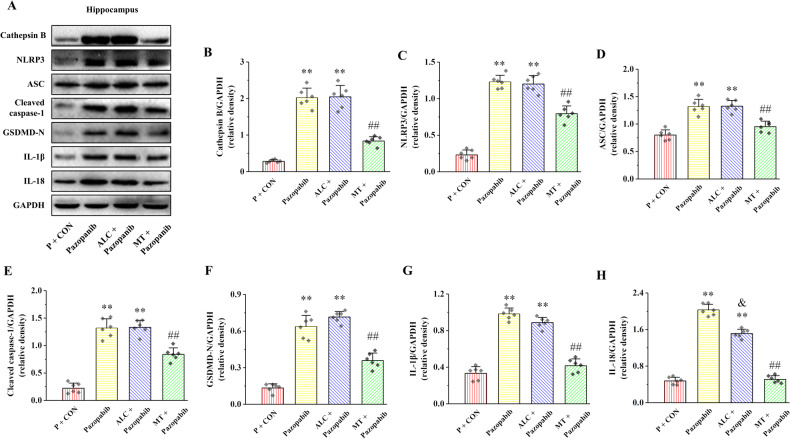
Effects of MT on hippocampal Cathepsin B expression, NLRP3 inflammasome activation and pyroptosis in rats induced by Pazopanib. **A** The relative protein expression of Cathepsin B, NLRP3, ASC, Cleaved caspase-1, GSDMD-N, IL-1β, and IL-18. **B**–**H** Proteins quantitative analysis of Cathepsin B, NLRP3, ASC, Cleaved caspase-1, GSDMD-N, IL-1β, and IL-18. All data are presented as the mean ± SD (*n* = 6).^*^P < 0.05 and ^**^P < 0.01 versus P + CON group. ^##^*P* < 0.01 versus Pazopanib group. ^&^*P* < 0.05 versus ALC + Pazopanib group.

To clarify whether MT relieve hippocampal pyroptosis though inhibiting Cathepsin B/NLRP3 signaling pathway, Pazopanib was used to activate Cathepsin B. The proteins expression of Cathepsin B, NLRP3, ASC, Cleaved caspase-1, GSDMD-N, IL-1β, and IL-18 in Pazopanib and ALC + Pazopanib groups compared with *P* + CON group, whereas these proteins expression were clearly declined in MT + Pazopanib compared with ALC + Pazopanib group (Fig. 9A–H, *P* < 0.01). These results indicate that MT attenuates Pazopanib-induced pyroptosis in the hippocampus by inhibiting Cathepsin B/NLRP3 signaling pathway.

## Discussion

Chronic stress is one of the important pathogenic factors of depression [35]. Depression-like behaviors are closely related to chronic stress-induced hippocampal inflammation and pyroptosis [11]. Recent studies have shown that MT can improve hippocampal damage and depression-like behaviors caused by chronic stress [36]. However, the exact prevention mechanism and therapeutic targets of MT on hippocampal damage still need to be further elucidation. In this study, we demonstrate for the first time that MT ameliorates chronic stress-induced hippocampal damage and subsequent depression-like behaviors by inhibiting microglia pyroptosis. Furthermore, MT alleviates chronic stress-induced hippocampal microglia pyroptosis by inhibiting Cathepsin B/NLRP3 signaling pathway.

Overactivation of the HPA axis is also one of the important markers of stress response, leading to increased release of glucocorticoids [5]. In fact, CORT is widely used in the establishment of in vitro models of chronic stress [37, 38]. In this experiment, CORT decreased the cell viability of HAPI cells, while MT reversed this phenomenon. The cell membrane of pyroptosis cells was ruptured, and the ultrastructural changes such as swelling of cells and organelles, large vesicles on the plasma membrane, and vacuolization of organelles could be observed under transmission electron microscope [39]. In this study, MT improved the microstructural and ultrastructural damage of HAPI cells caused by CORT. However, the mechanism by which MT ameliorates cell damage remains unclear.

Recent studies have shown that pyroptosis is widely involved in various nervous system diseases [15]. Pyroptosis mediated by NLRP3 inflammasomes can activate GSDMD to the terminal fragment of GSDMD-N. The activated GSDMD-N can perforate the cell membrane, leading to cell swelling and rupture, accompanied by the release of a large number of inflammatory cytokines [40, 41]. In this study, flow cytometry showed that HAPI cells experienced programmed death characterized by cell membrane rupture, including pyroptosis, programmed necrosis, and late apoptosis, but not early apoptosis. Studies have shown that Z-YVAD-FMK is a caspase-1 inhibitor, which is widely used to inhibit cells pyroptosis [42]. To determine whether pyroptosis is involved in CORT-induced HAPI cells death, Z-YVAD-FMK was used in the present study. Our results showed that Z-YVAD-FMK significantly reduced CORT-induced pyroptosis key proteins expression such as Cleaved caspase-1 and GSDMD-N, inflammatory cytokine release, and HAPI cells death, suggesting that pyroptosis was largely involved in CORT-induced HAPI cell death. MT can relieve CORT-induced HAPI cells pyroptosis by inhibiting NLRP3 inflammasome activation. Consistent with the results of the present study, MT attenuates LPS-induced NLRP3 activation in microglia [43]. These results suggest that MT alleviated HAPI cells death at least in part due to its inhibition of NLRP3-mediated pyroptosis.

In order to further explore the mechanism of MT improving microglia pyroptosis and potential targets of MT, PPI network analysis and a molecular docking model were used to predict the potential regulatory protein of NLRP3. Our results suggest that Cathepsin B is a potential regulator of NLRP3. Moreover, MT inhibited the mRNA and protein expression of Cathepsin B induced by CORT. Meanwhile, a molecular docking model of MT and Cathepsin B was constructed. In our study, MT was able to bind with Cathepsin B with a binding-energy value of −5.68 kcal/mol. These results indicate that MT can be naturally dockable with Cathepsin B, and the docking effect is good (when the binding-energy value is less than −1.2 kcal/mol) [25]. Our results show that MT can naturally dock with Cathepsin B and the docking effect is good. These results suggest that MT ameliorates NLRP3-mediated pyroptosis most likely through modulation of Cathepsin B.

Studies have shown that Cathepsin B gene knockdown can exert a neuroprotective effect, ameliorating behavioral deficits and pathological damage in neurological disease models [44]. Moreover, the leakage of Cathepsin B is an upstream event of NLRP3 inflammasome activation and subsequent pyroptosis [44]. The inhibition of Cathepsin B/NLRP3 signaling pathway has been reported to play an anti-inflammatory role in the reduction of kidney injury by hydroxychloroquine [45]. To elucidate the role of Cathepsin B/NLRP3 signaling pathway in CORT-induced HAPI cells pyroptosis, CA-074Me was used to inhibit Cathepsin B expression. Meanwhile, NLRP3 was knocked out using the CRISPR/Cas9 gene-editing technique. In this study, both Cathepsin B inhibition and NLRP3 knockout inhibited HAPI cells pyroptosis induced by CORT. Notably, inhibition of Cathepsin B expression greatly reduced NLRP3 expression, and although NLRP3 knockdown also reduced Cathepsin B expression, the effect was not satisfactory in comparison. The effect of NLRP3 knockdown on Cathepsin B expression may be related to the negative feedback after the reduction of pyroptosis. These results suggest that the inhibition of Cathepsin B/NLRP3 signaling pathway also plays a therapeutic role in CORT-induced HAPI cell pyroptosis.

To further validate the inhibition of MT on Cathepsin B/NLRP3 signaling pathway, we treated cells with Pazopanib, an agonist of Cathepsin B. Previous studies have shown that Pazopanib is also an effective selective pyroptosis activator [46]. Our results showed that Z-YVAD-FMK significantly reduced Pazopanib-induced pyroptosis key proteins microglia and GSDMD-N expression, inflammatory cytokine release, and cell death, suggesting that Pazopanib-induced HAPI cell pyroptosis. However, MT can relieve Pazopanib-induced HAPI cells pyroptosis by inhibiting Cathepsin B/NLRP3 signaling pathway. The above results indicate that MT alleviated Pazopanib-induced HAPI cell pyroptosis through inhibiting Cathepsin B/NLRP3 signaling pathway.

Previous research in our group has found that MT can improve chronic stress-indcued depression-like behaviors in rats by alleviating hippocampal damage [25]. Similarly, MT can reverse depression-like behaviors in adult mice exposed to chronic mild stress [47]. However, it is unclear whether MT improves microglia pyroptosis by inhibiting the Cathepsin B/NLRP3 pathway, thereby alleviating hippocampal injury and subsequent depression-like behaviors. Consistent with the results of in vitro experiments, MT inhibited the expression of pyroptosis and Cathepsin B/NLRP3 signaling pathway-related proteins. Moreover, the immunofluorescence triple staining results showed that key pyroptosis proteins were co-localized with microglia. These results indicate that MT improves hippocampal microglia pyroptosis through inhibiting Cathepsin B/NLRP3 signaling pathway.

To investigate whether hippocampal pyroptosis is involved in inducing depression-like behaviors of CRS rats. An oral active selective caspase-1 inhibitor VX-765, which is widely used to block pyroptosis [48], was used in this study. Our results showed that VX-765 improved hippocampal pyroptosis and depression-like behaviors in CRS rats. These results suggest that chronic stress-induced behavioral abnormalities are closely related to hippocampal pyroptosis in rats. Therefore, MT can alleviate hippocampal pyroptosis by inhibiting Cathepsin B/NLRP3 signaling pathway, thereby improving the depression-like behaviors of chronic stress rats.

Nitroxoline [49] and MCC950 [50] inhibitors of Cathepsin B and NLRP3, respectively, were used to further validated the role of Cathepsin B/NLRP3 signaling pathway in chronic stress-induced hippocampal pyroptosis in the current study. Consistent with in vitro experiments, both Nitroxoline and MCC950 were inhibited pyroptosis in the hippocampus. These results suggest that inhibition of Cathepsin B/NLRP3 signaling pathway can alleviate chronic stress-induced hippocampal pyroptosis in rats. Meanwhile, to further verify whether MT can improve hippocampal pyroptosis by inhibiting Cathepsin B/NLRP3 signaling pathway, MT was used to inhibit Pazopanib-induced hippocampal pyroptosis in vivo. Our results suggest that MT can improve hippocampal pyroptosis by inhibiting activation of Cathepsin B/NLRP3 signaling pathway induced by Pazopanib. The above results again confirm that MT can improve hippocampal pyroptosis in chronic stress rats by inhibiting Cathepsin B/NLRP3 signaling pathway.

However, there are still some limitations in this study. MT can also significantly inhibit the gene expression of other NLRP3 regulatory proteins, such as NEK7 and P2rx7. Therefore, the role of NEK7 and P2rx7 in MT improvement of chronic stress-induced hippocampal injury needs to be further elucidated.

In conclusion, this study used in vivo and in vitro experiments to relatively fully reveal and demonstrate the protective mechanism of MT in alleviating chronic stress-induced hippocampal injury and subsequent depression-like behaviors in rats. Specifically, MT alleviates chronic stress-induced hippocampal damage and subsequent depression-like behaviors by inhibiting microglia pyroptosis. In terms of molecular mechanism, MT improves chronic stress-induced microglia pyroptosis by inhibiting Cathepsin B/NLRP3 signaling pathway. This study provides solid evidence for the prevention and treatment of chronic stress encephalopathy with MT and provides a potential target for the development of novel antidepressant drugs.

### Supplementary information

Figure A.1

## Data Availability

The data that support the findings of this study are available from the corresponding author upon reasonable request.
