# Supplementary material for: Melatonin alleviates chronic stress-induced hippocampal microglia pyroptosis and subsequent depression-like behaviors by inhibiting Cathepsin B/NLRP3 signaling pathway in rats
Source: Transl Psychiatry. 2024 Mar 27;14:166. doi: 10.1038/s41398-024-02887-y (PMC10973390; doi:10.1038/s41398-024-02887-y)
Supplement: Supplementary file 1 — Figure A.1 [file 41398_2024_2887_MOESM1_ESM.docx]

**
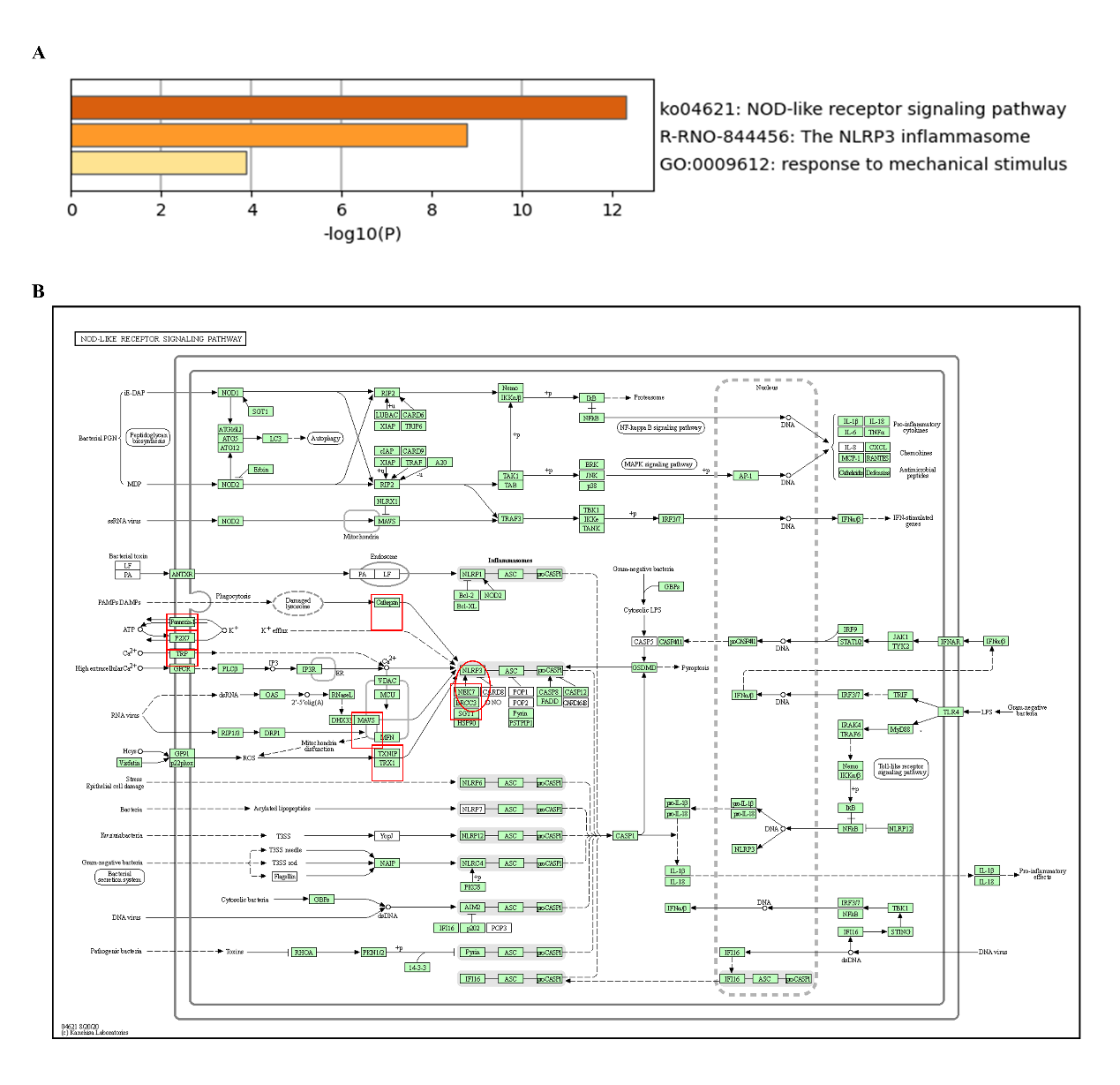
**

**Fig. A.1 The KEGG pathway enrichment analysis of Panx1, P2rx7, Mavs, Txnip, Cathepsin B and Nek7.** (A) The Gene Ontology term enrichment analysis. (B) The KEGG pathway map of NOD-like receptor signaling pathway.
